# People with type 2 diabetes struggling for self‐management: A part study from the randomized controlled trial in RENEWING HEALTH

**DOI:** 10.1002/nop2.293

**Published:** 2019-05-23

**Authors:** Lis Ribu, Marit Rønnevig, Juliet Corbin

**Affiliations:** ^1^ Department of Nursing and Health Promotion, Faculty of Health Sciences OsloMet—Oslo Metropolitan University Oslo Norway

**Keywords:** diabetes, health, nurses, nursing, RCT

## Abstract

**Aim:**

To develop a theoretical explanation for the daily life problems and challenges perceived by those living with type 2 diabetes.

**Design and methods:**

We used a grounded theory approach with a constant comparative method to discover a framework with the core concept of struggling between “ought to do” and “want to do” and related concepts.

**Results:**

The struggle to self‐manage and maintain new habits can be more or less difficult depending on the patient's perceived conditions. We identified three situations illustrated in a diagram: one where there is less struggle to let go of old habits, a second where there is more of a struggle to balance between what individuals want to do and what they ought to do and a third where they are giving up struggling. Study findings show that healthcare personnel must consciously seek to understand how patients perceive their own situation.

## 1 INTRODUCTION

The number of people who develop type 2 diabetes is rising globally, with two‐thirds of diabetes patients being of working age (International Diabetes Federation, 2017). Type 2 diabetes, consistent with other chronic conditions, requires “unending work and care” to control symptoms and to prevent complications, as earlier described (Corbin & Strauss, 1988), and patients often experience treatment burden due to the challenges and stresses caused by this complex disease (Eton et al., 2015). In addition, type 2 diabetes places considerable demand on healthcare systems in terms of types of services needed, manpower and management costs (da Rocha Fernandes et al., 2016). Those with type 2 diabetes are expected to be actively involved in decision‐making regarding their own treatment and to carrying out home‐based self‐management (American Diabetes Association, 2017; The Norwegian Directorate of Health, 2017). Self‐management has been defined as “the individual's ability to manage the symptoms, physical and psychosocial consequences and lifestyle changes inherent in living with chronic illness” (Barlow, Wright, Sheasby, Turner, & Hainsworth, 2002).

## 2 BACKGROUND

Although people with diabetes strive to make behaviour changes through self‐management, in Norway, only 55% among those with type 2 diabetes attain their long‐term blood glucose treatment goals (Mouland, 2014). Some of the conditions that affect a person's ability to carry out illness self‐management include health status, amounts and types of resources available, environmental characteristics, level of knowledge and access to healthcare systems (Schulman‐Green, Jaser, Park, & Whittemore, 2016). More specific has lack of knowledge about diet, frustration about an inexplicable disease progression and poor glycemic control, earlier been described (Nagelkerk, Reick, & Meengs, 2006). Further, the patients' reluctance to discuss poor self‐management can hinder the communication between the patients and the physician, as well as the physicians' lacking strategies to handle such problems (Ritholz, Beverly, Brooks, Abrahamson, & Weinger, 2014). Earlier research has found that although HCPs are supportive, many patients indicate that they are not discussing key aspects of their care such as anxieties or nutrition, both of importance for the patients' possibility to self‐manage (Nicolucci et al., 2013). It has also been described how some patients perceive that their self is under attack when healthcare personnel (HCP) inform them that they are failing because their blood sugar levels are too high. Because it is the patients' perceptions of the situation that determine how they act, HCPs must consider patients' feelings as they counsel them about such matters (Gomersall, Madill, & Summers, 2011). Family motivation is another important facilitator for self‐management (Ong, Chua, & Ng, 2014).

It has earlier been described how communication, education and support from others in general may influence self‐management and how the ability to self‐manage is a dynamic and evolutionary process that varies from person to person. Individualized support specific for the patient is therefore important (Wilkinson, Whitehead, & Ritchie, 2014), and ongoing education and support also include a healthcare team collaborating with the patient in setting goals and initiate proper interventions (Powers et al., 2015). Psychosocial issues are especially important in lifestyle self‐management, and the assessment of the disease, affect and mood, quality of life, available resources, psychiatric history and patients' expectations to treatment and outcomes are recommended. It is recommended to include caregivers and family members in this assessment (American Diabetes Association, 2018).

It has also been described how professionals take a “disease over life” perspective, while patients just want “to live their life as well and normally” as possible. Research has shown how this conflict can be especially strong and disempowering for those with poor problem‐solving skills (Zoffmann & Kirkevold, 2005). Despite a relatively huge body of research in this area, we still lack a comprehensive understanding about why some persons do not reach their treatment goals while others succeed.

### 2.1 Aim

The aim of this study was to attain in‐depth knowledge of the persons' perceptions and responses to what was happening in their lives and to identify the conditions and social patterns that affect self‐management. Our research questions were *What are the conditions to which patients with type 2 diabetes perceive within the process of their illness and to which they respond? What are their responses (actions–interactions or strategies) to the identified conditions? What are the outcomes of their responses? How do patients perceive their situation?*

This qualitative study was part of a larger randomized controlled trial (RCT) that aimed to evaluate telemedicine self‐management tools, the details of which have been previously described (Holmen et al., 2014; Ribu et al., 2013; Torbjørnsen et al., 2014). In this study, we attained more in‐depth knowledge about the persons voluntarily participated in this study, according to their self‐management and daily living with type 2 diabetes.

## 3 DESIGN AND METHOD

In this presentation, we follow the recommendations from the Consolidated Criteria for Reporting Qualitative Research (COREQ) (Tong, Sainsbury, & Craig, 2007).

We used a qualitative research design with a grounded theory (GT) approach to derive inductively a theoretical explanation for the process of living with type 2 diabetes. GT has an interactionist perspective and is concerned with identifying the psychosocial processes that explain human behaviour in various situations (Corbin & Strauss, 2008, 2015). GT also gives a systematic set of techniques and procedures to identify concepts during the research process. This approach uses a constant comparison method to verify the importance of concepts identified during the research process and to develop the concepts' properties and dimensions (Corbin & Strauss, 2008, 2015).

### 3.1 Participants

The study sample was adults with type 2 diabetes aged ≥18 years, with HbA_1c_ level ≥ 7.1% and those who were able to complete questionnaires in Norwegian. Participants were recruited from the two intervention groups in the Norwegian three‐armed RCT of the EU project RENEWING HEALTH (RH) (*N* = 79). The details of the Norwegian study have been previously described (Ribu et al., 2013; Torbjørnsen et al., 2014; Holmen et al., 2014).

Study participants who agreed to follow‐up interviews when signing informed consent at initial enrolment were included. Further, the same participants were assessed for their eligibility consecutively when they had finished their year‐long participation in the original RCT. We conducted the interviews when the participants left the study, and the interviews continued until a degree of theoretical saturation was obtained. Of the first 50 participants eligible, 15 did not participate because they were in poor health, too busy, impossible to reach for an appointment, on vacation, etc. Of the remaining 35 willing to participate, seven ultimately declined to be interviewed and two became too ill to do so. We interviewed this remaining pool of 26 participants.

### 3.2 Data collection

We collected data from May 2012–March 2013. We conducted 26 face‐to‐face, open‐ended, in‐depth interviews in the participant's home, the interviewer's office or by telephone for those who lived far away (according to participants' preferences). All interviews were audiotape, but only 24 were analysed because of mechanical problems with the recorder during the interview process. All tapes were transcribed verbatim and analysed by two of the authors (MR & LR). The interviews ranged from 22–90 min (average 48 min). The participants gave one interview each, and we wrote memos after the interviews. The next interview was then built on experiences with the latter interview. We also returned to data to get more answers and information during the data analysis process.

A semi‐structured interview guide was used in the interview process, although participants were free to add new elements during the interview (Appendix 1). The guide contained open‐ended questions about (a) living with and managing their diabetes, (b) interactions with self and others (e.g., relatives, general practitioner, diabetes nurse) and (c) interactions with their environment in general and with technology specifically (i.e., technology described by Torbjørnsen, Ribu, Rønnevig, Grøttland, & Helseth, 2018). Data about the participants' acceptance with the technology will be published elsewhere (Torbjørnsen et al., 2018).

### 3.3 Data analysis

Consistent with GT, data analysis occurred concurrent with data collection and included writing memos on the comparative, summary and integrative types, that is, linking categories around a core category to form theory (Corbin & Strauss, 2015). The first step of analysis was open coding and writing memos (Table 1). Our unit of analysis was incidents sharing some common characteristics and not people. Incidents refer to happenings that lead to responses. We compared these incidents for conceptual similarities and differences both within and between interviews. Nearly all described how they tried to attain new and healthy habits and to adapt strategies to meet ever‐changing conditions and perceived risks. They described this as a constant battle, ongoing every day and the *struggling* came up as a useful higher‐level concept to describe their trying, failing, excuses and reattempts. The responses to these incidents led us towards the core category *struggling between want to do and ought to do*. This category was not so much about their diabetes, but about managing their conditions.

**Table 1:** One example of analysis related to control the blood sugar

Open coding (descriptive codes)	Participant 75 year, experienced a dangerous high blood sugar: “Then, it is one thing to do, and I was not interested in chewing tablets. I changed diet and took control. I have to take care of myself to be further alive” It is hard to accept that you cannot eat what you want. I always have to look back and scrutinize: what did I do to get this high blood sugar measure? My blood sugar is increasing and I am trying to have a healthy diet and to be in activity. The problem is that I do not recognize symptoms from my diabetes, so it is…as it is not there…, but the blood sugar measures show that it is present…(…). I have got diet advices in all directions. I skip out when it is far too much. Then, I am eating junk food and choosing bad solutions… I don't know why it is so difficult. My physical condition was good when I was young, now my weight is pending up and down, and I am trying to get control. My blood sugar is high		
Low‐level coding (concepts, categories)	They *know what they ought to do*, but for most of them, *doing it is difficult* They are *trying* to have a healthy diet; *working hard* to balance a silent diabetes with no symptoms and poor blood glucose measures; often *failing* in handling an incomprehensible blood sugar. Some are more or less *giving up*		
High‐level coding (category)	They are *struggling between what they “ought to do” *versus* what they “want to do”*		
Theoretical coding	“Situations” with three categories of struggling^a^		
	(1) less struggling	(2) considerable struggling	(3) some struggling, often giving up, and in need of tight support
Condition	Experience bodily symptoms of high blood sugar	No or few experiences of bodily symptoms of high blood sugar	Having a body that is always feeling tired due to high degree of underlying disease

a “Situations” are illustrated in Figure 1 and described in the end of the Result section in the text.

The second analysis step to make theory was axial coding using the analytic tool paradigm described by Corbin and Strauss (2008, 2015). Paradigm includes conditions, action–interactions and consequences or outcomes, and we used this tool to sort out and look for possible links between concepts (Corbin & Strauss, 2015). Conditions refer to the perceived reasons that the people give for why, when and how they responded (action–interaction) to an incident. Conditions can generate emotions such as resignation when failing in behaviour change, and their confidence in being able to changing habits becomes low. A change in conditions can lead to changes in the action–interaction. Action–interaction is the responses persons give to situations in their lives and, for example, how they manage a problem they have. Consequences are outcomes of these action–interactions to self or others and are actual or anticipated. At last in the result section, we describe the situations that may explain an action–interaction within the background of certain conditions and anticipated consequences, to explain why some people struggle more than others do. Thus, we linked the concepts and delineated the beginning of a theory according to Corbin's paradigm (theoretical coding) (Corbin & Strauss, 2015).

### 3.4 Ethical considerations

The Regional Committee for Medical and Health Research Ethics in Norway (REC no 2010/427) approved the study. Participants received both written and verbal information during the larger RCT, including assurances of full confidentiality and the right to withdraw from the study at any time without giving a reason for doing so. At that time, participants also consented to being contacted for follow‐up participation in an in‐depth interview after the RCT. When contacted for the interview, participants received detailed written and verbal information about the interview and were given general procedural information. After each interview, data were stored anonymously by replacing names with codes and tape recordings were stored in a locked safe.

## 4 RESULTS

*Struggling between “*want to do*” and “*ought to do*”* was the core concept that emerged from these data to explain the process by which people with type 2 diabetes attempt to manage their diabetes and their perceived conditions (Figure 1).

**Figure 1:**
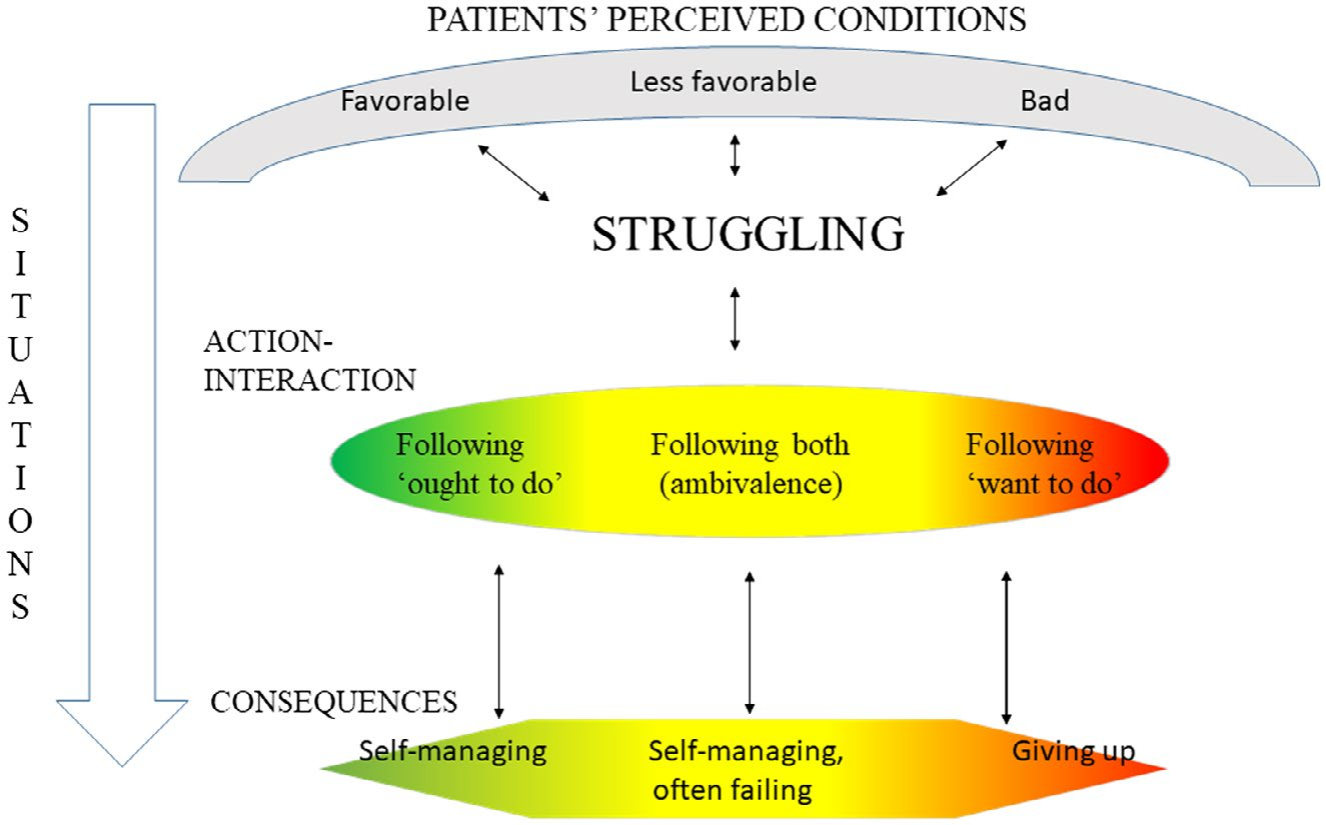
Struggling between “ought to do” and “want to do”

The idea behind diabetes management is to make healthy living behaviours into habits so that the struggle becomes easier and *the ought to* part of the struggle wins more often than the *want to* part. However, making the *struggle* easier and developing new habits are complicated because the struggle to develop and maintain healthy living habits occurs within a context of conditions that are *favourable*, *less favourable* or *bad*. These conditions can be of a personal or environmental nature. They can also vary in degree (favourable–bad) and dimension (e.g., low–high) and can either facilitate or constrain the individual's ability to handle challenges through strategies (action–interactions). Conditions that facilitate their ability to handle problems make it easier for patients to do what they *ought to do*, while conditions that constrain them make patients more likely to give up the *struggle* or give in to *want to*, because old habits are easier to carry out and give more perceived pleasure compared with new habits. It is, however, important to note that *want to do* and *ought to do* do not have to be mutually exclusive. Some persons *want to do* what they *ought to do*. They want to eat nutrient‐dense foods, exercise and privilege their health. This group is categorized as *ought to do* in the present model. The preferred *outcome* is to *self‐manage*. Some experience failing, while others find that they are giving up. In Figure 1, green illustrates the first group (no danger/walk), yellow illustrates the group struggling the most (alarm), and red illustrates the group that has given up struggling (danger).

### 4.1 Patients' perceived conditions

The conditions identified by the participants in our sample as the context for action–interaction are described in Table 2. The patients describe how differences in each condition vary in degree from *favourable* to *bad* and contribute to greater or less *struggle* in performing activities that they are supposed to do to *self‐manage* in situations.

**Table 2:** Conditions. These vary in degree from high to low

Conditions	Definitions
Health competence	Refers to persons' ability to manage their medical condition. Competence requires a certain level of knowledge about the condition and the regimen needed to control it. Competence also refers to, the ability to read body cues and carry out tests that indicate high or low blood sugar and the ability to adjust daily regimens based on test results
Motivation	Refers to willingness to make the changes in lifestyle required to manage their condition. High motivation requires an acceptance of having diabetes
Confidence	Refers to the previous experiences with handling difficult situations and making change
Patient burden	Refers to severity of the disease, presence of complications and/or co‐morbidities, and/or being overweight (a contributing factor). For some persons, there are no symptoms when blood sugar is high making it difficult to manage or accept they have diabetes
Lifestyle	Refers to eating the foods recommended and avoiding the types and amounts of foods that tend to increase weight and/or raise blood sugars Refers to an understanding of the importance of including exercise into daily life and the mental will and physical ability to carry it out
Environmental factors	Refers to living conditions that are conducive to having healthy diet and carrying out physical activities. It includes having access to safe outdoor places and accommodating families and working conditions
Resources	Refers to the presence or absence of the financial ability as well as access to treatment, social support and transportation to and from healthcare facilities and places to exercise
Supportive professional relationship	Refers to the ability to develop and maintain open and honest communications between patient and health professionals and having relationships based on mutual respect and consideration of patients' life situations
Family and friends support	Refers to how person with diabetes and family and friends relate to each other in regard to the diabetes and families and friends' willingness to make the necessary adjustments in their lives to accommodate to the special needs of the persons with diabetes

### 4.2 Action–Interaction

Even the most highly motivated may find it difficult to attain and maintain change when faced with difficult conditions. This is where action–interactions (strategies) come in. Both *letting go of old habits* and *taking on new habits* require individuals to take an active role in changing the conditions that might prevent them from succeeding. In our analyses, we identified three categories of such struggling.

Those following what they *ought to do* in their *struggle* used strategies such as “making routines”; “making rules for action”; “using rewards to keep motivation”; “educating family and friends” about their diabetes treatment regimen; “working together with health professionals” and “involved in management”. For example, a major strategy for maintaining a healthy diet was avoiding temptation through rules such as “not keeping forbidden food at home”. “If I have two litres of ice cream, it will be gone within two hours.” There were times when individuals allowed themselves to indulge in foods that were not on their diet, but this was a transient event. They might restrict food intake during the week to allow more in the weekend.

Those following what they *want to do* were “not accepting that change is necessary” and some were “giving up” the fight. Furthermore, they had “no routines” and many were “ignoring their disease”. Many lacked energy to take care of themselves. “I am told to exercise, but I am so tired. It is as if I do not care. I want to do some exercise, but I do not bother. I do not get started.”

Those in the largest group, who followed both what they *ought to do* and what they *want to do*, action often failed to make changes. They knew *what to do* but expressed that doing it was very difficult. Strategies among individuals in this group differed from those in the other two groups in that they made use of environmental and other conditions to “explain” and “excuse” their failure. For example, patients might tell themselves, or their HCPs, that they could not exercise because foot ulcers prevented them from doing so, rather than looking for alternative ways to exercise to accommodate their disability, such as performing chair‐based activities rather than strenuous walking.

Many asked for another support than that they received: “I wish I had someone to support me by showing interest by asking such things as: How is it going? Are you taking your walks?”

### 4.3 Consequences

The patients perceived that *self‐managing* of their diabetes was a desired goal. They described how they *struggled* to incorporate diabetes into their daily lives by *self‐managing* through activities such as diet and exercise, medication and following routines. Because accommodations must be lifelong and life circumstances or conditions tend to change over time, this is an ongoing process. Only the individual can *self‐manage*, although it helps to have support from relatives and healthcare personnel. *Self‐management* manifests in our analysis as balancing *ought to do* and *want to do*, in a constant *struggle* to live a healthy life. Many of the patients in our study were failing in their *struggle,* and some were giving up (Figure 1).

### 4.4 Situations

This research has identified three categories of struggling, which lead to three situations explaining why some individuals struggle more than others: (1) situations where there was *less struggle*; (2) situations where patients had *more of a struggle* and tended to vacillate between doing what they *ought to do* and what they *want to do*; and (3) situations where patients *gave up the struggle*. The section below examines each of these three situations in greater depth, based on our analysis.

In Situation 1, where there was *less struggle* for patients to manage their diabetes, conditions were more favourable to change, or participants had a repertoire of strategies for “working around” conditions that might otherwise stand in the way of making change. They perceived the situation as necessary to manage:

I had many unhealthy habits. When I was diagnosed with type 2 diabetes, I realized that I had to tighten up myself. I started thinking differently, I knew I had to postpone complications.

People who followed the regimen were those who remained in constant dialogue with themselves and others to assess situations and to work out ways of managing. “Slowly but surely, I change my habits. I am still smoking, but I have agreed with the GP that it has to be coming.”

There was no discrepancy between what the HCPs wanted participants to do and what the participants themselves wanted to do. A major strategy used by people in this group was to establish or maintain a “working” relationship with their HCP. “You must have a good relationship with your GP, otherwise you have a problem”. Some in this group even changed their GPs when they felt that they did not receive the amount and type of support that they needed.

In addition, there were few environmental and family conditions preventing them from making changes to their habits. Achieving and maintaining change was, however, not easy for this group either, and there were still obstacles to doing always what they *ought to do*.

These patients had the ability to read body cues indicating when their blood sugar levels were too high or low. Further, they kept track of their blood sugar levels through “frequently monitoring their blood glucose” and “comparing monitoring results against their diet and activity”. They worked at maintaining a positive attitude by telling themselves “you will make it through this” and “you have to go on with life.” They found ways to include exercise in their daily routines by walking the dog or connecting exercise with doing household chores. Some attended groups that focused on physical training (e.g., walking and swimming).

People in this group developed new attitudes regarding food, thinking of it as “a treatment and kind of medication.” They learned to prepare and eat healthy foods, for example, “buying fresh food and making it from scratch.”

These persons seem to be more accepting of their disease saying that “despite their diabetes,” they were happy with life. They recognized that they “had to find their own way and take care of themselves after all.”

In Situation 2, participants had *more of a struggle* to develop and sustain new habits. They tended to balance between doing what they *ought to do* and doing what they *wanted to do*. It seems that they had fewer strategies for managing difficult situations:

I have found a key (blood sugar) value that is working for me. However, I lose focus halfway and I am easily distracted. There is also so much to do that is more fun than doing exercise.

Because of co‐morbidities or complications from their diabetes, physical activity was reported to be difficult, including finding appropriate physical environments that would enable them to perform the activities that they were able to do. Many had never exercised and did not like to do so. Furthermore, this group perceived a lack of information about how and what to eat. They found it too stressful and time‐consuming to read the ingredients on tins and boxes of food to find out what they contained. One participant reported as follows:

I measure my blood sugar when I get home to see what it is. When I see how high the blood sugar level is, I realize I should not have eaten what I did. However, what should I have eaten?

Relatives and friends of those in this group were not always supportive. Families were unwilling to let go of their old eating habits. “Usually I manage OK, but I get tired of making two dinners. My family will not eat the same as me.”

Many people in this group perceived that they did not have competent and caring support from their HCPs. A diabetes nurse was unavailable to them because they were not ill enough. Dialogue with their GPs was often limited to discussing biomedical measures. There was little or no time or interest on the part of their GPs to listen to the patients' experiences with everyday life problems.

Some participants in this group acquired knowledge about diabetes and diabetes management at the time of their diagnosis and followed the diabetic guidelines at first. However, after a while, their motivation diminished and they fell back into old habits of eating or not exercising:

Many participants in this group experienced feelings of guilt and anger:

I try the best I can. However, I do not always succeed. Sometimes I get so angry with myself because I have eaten something that I should not have eaten. Afterwards I have regret.

In Situation 3, participants “gave up the struggle” to develop or maintain new habits necessary to keep their blood sugar levels stable and within a range necessary to maintain optimum control over their type 2 diabetes. It is important to keep in mind that conditions can change and that any major change may bring about a “renewed” *struggle* between *ought to do* and *want to do*.

Some in our study felt that the conditions under which they were living (home life, disabilities) were incompatible with developing and maintaining a healthy lifestyle. They gave up when *ought to do* was too hard and more often than not followed the *want to* path. Many of these participants expressed knowledge about what they were supposed to do but lacked strategies for making such changes. It was a type of downward spiral where they lacked energy and continuously felt tired, which they described in terms of “fluctuating or elevated blood sugar level,” “low energy” and “fatigue.” The development of diabetic complications and/or co‐morbidities made it even more difficult for them:

I would like someone to come and take me by the ears until I have started exercising. We know we must. We were good at it for a while, but then poor circulation in the legs put an end to that.

The main strategy was to follow what they *want to do:* “I do nothing to reduce weight. I take it as it comes.”

These individuals indicated that they had a no supportive relationship with their GP and that they experienced a lot of stress by “looking for a new available GP.” There was a gliding transition between the strategies in situations 1 and 2, but the negative strategies, or lack of strategies, used by patients in group 3 blocked their ability to change habits.

The consequences of not manage to take care of themselves and giving up the struggle might be permanent or temporary. If temporary, a person might get back into the struggle and, for example, gain better control of blood sugar levels and disease course.

## 5 DISCUSSION

This study gives a novel explanation of the problems and challenges perceived by people with type 2 diabetes who are struggling to make lifestyle changes. Our findings suggest that those living under a less favourable situation struggle the most and for them, living with diabetes was a continuous conflict between *want to do* and *ought to do.*

One important finding in this study is that many of the participants struggling the most perceived a need for more support than they received from their HCPs, and the support that they received from their GPs was perceived as advice and medical treatment rather than respect and understanding. No time was allotted during consultations for discussing everyday life challenges related to their disease. It has earlier been described how HCPs also are responsible for involving the patients in decisions and not necessary expect them to follow their advice (Zoffmann et al., 2016).

Yet, not everyone in our study was willing to discuss their situation with their HCPs. Previous research has identified and examined factors associated with patient reluctance to discuss self‐management in a treatment relationship (Beverly et al., 2012; Ritholz et al., 2014). Reluctant patients reported that they did not want to disappoint their doctors or to be judged for not managing. Many reported shame, guilt and embarrassment, consistent with the patients in our study. Such relationships may be a barrier to learning and should be raised and discussed (Beverly et al., 2012; Ritholz et al., 2014).

The patterns described by the persons in our study can facilitate the HCP's understanding of their situation. It has earlier been described in a model for illness integration support for people with type 2 diabetes, how active listening and a focus on emotional and existential issues is factors facilitating persons' self‐management (Hörnsten, Jutterström, Audulv, & Lundman, 2011). Further, the support to these patients should be respectful and allow for the persons preferences and needs and the persons' values should guide decisions (Powers et al., 2015). The HCP must also pay attention to different point of views between patients and professionals (Zoffmann et al., 2016). In our study, some persons did change their GP, due to confusing support or too strict advices, but the situation was seldom described as better with a “new” GP. The life with diabetes can be troublesome for many, due to several recommendations to follow, and as such, we are questioning whether the guidelines and/or the healthcare personnel have failed to meet these persons in their situations. Persons with diabetes struggle most of the time alone in their homes, to develop their own strategies for self‐managing and living their life, and more attention could therefore be given to both the persons' themselves and their context and also to the civil society. Multisectorial and population‐based approaches are recommended against overweight and obesity and other risk factors such as an unhealthy diet or little physical activity. Supportive environments and support from the highest level of government are necessary and to involve different sectors in this work (e.g., producers of food). Thus, a broader approach to these problems is necessary (WHO, 2016).

The persons in need of self‐management support are, however, not a homogeneous group, and we have categorized the persons in our study into three groups due to their perceived situations. Earlier research has shown that those living with diabetes and improved their outcomes were more engaged and had higher self‐efficacy and were more likely to adopt newly learned skills, than disempowered persons with worse health and few resources and with lower positive and active engagement in life. The latter had, however, the potential to increase both health and social inequalities, when HCPs were using this knowledge to encourage these persons to participate in lifestyle programs (Packer et al., 2012). Particular considerations should, however, be given to populations of lower socioeconomic status (WHO, 2016). In our study, we found, for example, that those who were receivers of minimum state pension perceived economical restrictions to do what they wanted to do.

Our study has identified a broader range of conditions and other issues perceived by patients influencing their opportunity to self‐manage. These issues can be used to a further development of a checklist or patient‐reported outcome measure (PROM) (Nelson et al., 2015), as a basis for creating a meaningful dialogue by listening to the patients. The knowledge gained from such instruments could ensure that HCPs develop skills necessary for guiding patients' needs to self‐manage their diabetes in their daily life. Such knowledge would also be valuable in designing interventions for those with type 2 diabetes.

Our findings are also in line with a metasynthesis of previous research of persons with long‐term illness, largely adults with diabetes and cardiovascular disease, that gave a detailed description of negative (barriers) and positive (facilitators) factors influencing self‐management (Schulman‐Green et al., 2016). The authors described interaction of factors forming a “factor‐profile” as helpful in determining needed self‐management interventions. In our study, we have identified three groups and analysed how more or less favourable situations affect their self‐management. The shifting conditions and complex situations that those with type 2 diabetes perceive make it important to pay more attention to these groups, the individuals within the groups and their overall and ever‐changing situation.

### 5.1 Strengths and limitations

We did not conduct a theoretical sampling according to Corbin and Strauss' methods (2015), because our participants were drawn from a larger RCT on lifestyle change, where volunteers were provided with smartphones with a diabetes diary app for self‐management. Therefore, it is possible that our participants were especially motivated to change. On the other hand, this sample was heterogeneous based on sociodemographic and clinical variables, because of successful randomization into the RCT. Despite this, not using theoretical sampling is a limitation in a GT‐based study. Theoretical sampling is used to ensure that the theory is developed iteratively; drawing on any relevant data sources needed to understand further the factors influencing a particular event or concept. In the present study, however, we conducted interviews that built on our previous experiences and returned to the data to answer on our questions. We finished when saturation was attained and no new concepts emerged.

A strength of the study was that we discovered that those who made up the largest group had the greatest struggle with achieving and maintaining behaviour change. This finding is in line with earlier research (Gomersall et al., 2011; Schulman‐Green et al., 2016; Zoffmann & Kirkevold, 2005) and confirms and expands findings from RH. The latter shows that many with type 2 diabetes are in a pre‐action phase in terms of behaviour change (Holmen et al., 2016).

## 6 CONCLUSION

Our findings represent a broad range of patterns perceived by persons living with type 2 diabetes, and a major study finding is that persons in each of the three groups needed different approaches from their HCPs because of the heterogeneous conditions under which they were living. Nurses and other HCPs need to assess individuals based on their unique struggles. Those who struggle the least need checking and supporting. The largest group, those who struggle the most, need help in developing strategies. Those who have given up need sophisticated individualized support and tighter follow‐up compared with those in the other groups.

It should not be expected that HCPs will find solutions to every patient's self‐management challenge, but there are many ways to support such patients. Different approaches merit further investigation.

## CONFLICT OF INTEREST

No conflict of declaration.

## AUTHOR CONTRIBUTION

Authors MR and LR analysed and reanalysed all of the interviews to find their essential concepts, discussed the coded data and resolved discrepancies through consensus. JC participated in some of these discussions and supervised the use of GT. Authors MR and LR wrote the paper together, and JC looked over the paper.

## APPENDIX 1

### INTERVIEW GUIDE

Tell me about your experiences about living with diabetes. Please tell me how you look at it. Start where you wish.

How do you experience the demands from living with a “24‐hr disease”?

What are the greatest challenges?

How do you experience the management that you receive?

How do you self‐manage?

How do you experience your interaction with others (nurse, general practitioner, other healthcare personnel, relatives, others)?

How is your motivation for changing lifestyle?

Supplementing questions:

Can you tell me more about that?

What do you do with that?

What did you think about then?

Could you have done something else?
